# Being a caregiver of a Behçet’s syndrome patient: challenges and perspectives during a complex journey

**DOI:** 10.1186/s13023-021-02070-2

**Published:** 2021-10-18

**Authors:** Rosaria Talarico, Diana Marinello, Arianna Manzo, Sara Cannizzo, Ilaria Palla, Simone Ticciati, Andrea Gaglioti, Leopoldo Trieste, Lorenzo Pisa, Luciano Badalamenti, Girolamo Randisi, Alessandra Del Bianco, Valentina Lorenzoni, Giuseppe Turchetti, Marta Mosca

**Affiliations:** 1 Rheumatology Unit, Azienda Ospedaliero Universitaria Pisana, Via Roma 67, 56126 Pisa, Italy; 2 Psychotherapist, Translational Analyst, Rome, Italy; 3 Institute of Management, Scuola Superiore Sant’Anna, Pisa, Italy; 4 Associazione S.I.M.B.A (Associazione Italiana Sindrome E Malattia Di Behçet), Pontedera, Italy

## Abstract

**Background:**

As often seen in many chronic diseases, the disease impact on patients also induces a significant impact on the quality of life (QoL) of caregivers. Caregivers are the ones who are really willing to offer care in the general approach of many aspects of the disease, including the awareness of the diseases itself, the daily management of therapy, and all the potential challenges that living with a chronic disease can include. The main objectives of the study were to explore the perspectives and views of caregivers of Behçet’s syndrome (BS) patients, to study their level of awareness on the disease and the impact that BS may have on their lives by means of a survey co-designed with caregivers and patients with this purpose. A survey was entirely co-designed with a panel of caregivers of patients living with BS patients.

**Results:**

Results show that BS caregivers organise their life according to the needs of the patient, that they (79%) considered themselves as helpful for the patient and 53% of them replied that they can freely express their emotions. Notably, 70% and 68% of the respondents reported they renounced with a variable frequency to sexual relationships due to concerns regarding the health of the partner or to the partner’s illness, respectively. The majority (79%) of respondents indicated that they are familiar with the treatment taken by the patients and that 68% deal with the administration of some medicines. In terms of awareness, a good percentage (64%) of respondents reported to understand the illness and, in terms of education, 68% of participants are willing to take part in training programmes dedicated to BS.

**Conclusions:**

The results of this survey contribute to provide new information on BS caregivers and on their important role, and to identify areas in which new initiatives could provide BS caregivers (and therefore patients) with tools and knowledge that can empower them in reducing the burden of the disease on their lives, on families, and on the patient.

## Introduction

Behçet’s syndrome (BS) is a systemic, chronic relapsing vasculitis, characterized by recurrent orogenital ulcers, ocular inflammation and skin manifestations; besides the typical manifestations, articular, vascular, gastroenteric and neurological involvement may also occur [1]. BS is characterized by a variable spectrum of disease profile; while prevalent muco-cutaneous involvement and arthritis represent the main clinical features in patients with a benign disease subset, there are other patients who potentially develop sight or life-threatening manifestations, due to ocular, neurological or major vascular involvement [2]. The relapsing nature of the disease can determine exacerbations and remission of symptoms over time; moreover, being free of major organ complications in the first years is not necessarily a sign of a favourable outcome, since about one third of patients may experience the development of de novo major involvement during the course of BS [3]. Various demographic factors are considered predictable of poor outcome in the short and long-term, such as age at disease onset, duration of disease or gender. In fact, younger male patients are generally more suitable to have a more severe disease, due to an increased frequency both of morbidity and mortality, related to ocular, vascular and neurological involvement. Taking into account all these elements, it clearly appears how a very careful and tight control is strongly recommended in BS patients, in order to manage in the most appropriate way the therapeutical approach according to disease activity [4, 5]. The chronic characteristics of the disease are strongly associated with a significant limitation of the daily activities as well as a potential negative impact on relationships with other people. Several data from the literature have demonstrated a strong relationship between disease activity and impact on the quality of life (QoL), also comparing the QoL of BS patients with other patients’ groups [6, 7]. Moreover, a high frequency of psychiatric disorders has been reported in BS patients, peculiarly represented by bipolar disorders and strictly related to the disease activity [8–11]. Therefore, the chronicity of BS features, together with many other different elements, may contribute to important challenges in the BS patient’s daily life. As often seen in many chronic and rheumatic diseases, the disease impact on patients can also significantly affect the daily life and the QoL of caregivers [12–19]. Family (Informal) Caregiver can be defined as “ any relative, partner, friend or neighbour who has a significant personal relationship with, and provides a broad range of assistance for, an older person or an adult with a chronic or disabling condition. These individuals may be primary or secondary caregivers and live with, or separately from, the person receiving care” [20]. Caregivers are, in fact, the ones who are really willing to offer care in the general approach of many aspects of the disease, including the awareness of the diseases itself, the daily management of therapy and all the potential challenges that living with a chronic disease can include. For this reason, it is particularly crucial to give voice to caregivers’ perspectives and points of view, especially in a relapsing and peculiar disease like BS, especially considering that these data were not available so far on BS. Thus, the aim of this work was to explore for the first time the perspectives of caregivers of BS patients, to assess their knowledge of the disease, their needs and the impact that BS has on their lives.

### Objectives

The main objectives of the study were to explore the perspectives and views of caregivers of BS patients, to study their level of awareness on the disease and the impact that BS may have on their lives by means of a survey co-designed with caregivers and patients with this purpose.

## Methods

In order to collect the perspectives and views of caregivers of BS patients, a survey was created. The survey was entirely co-designed in Italian with a panel of patients and caregivers of patients living with BS. In particular, the Italian patients’ association for Behçet’s disease, SIMBA OdV, supported the identification of 3 caregivers that were partners of patients living with BS and 4 BS patients that were then involved in the panel aimed at designing the questionnaire. The co-design panel gathered face to face in an interactive workshop and via email exchanges that were coordinated by a clinician expert in BS. A first version of the survey was drafted by an expert clinician and by a patient engagement manager and the draft was shared during the workshop. The survey was assessed by the panel of caregivers that reviewed the questions, the possible answers and proposed new questions to be added. After the workshop, the expert clinician and the patient engagement manager collected the inputs received and prepared a refined version of the survey that included all the comments and the proposals suggested. The refinement process included also a test phase to check the understandability, accessibility and actual functioning of the questionnaire. The final version of the survey was then approved by formal agreement of the panel of caregivers and the survey was uploaded into the online survey platform EU Survey [21]. An introductory text was also co-designed in order to provide information on the scope of the survey, on how it was developed and on the time needed to complete it. Participation to the questionnaire was voluntary and anonymous and they were asked for their consent to analyse their anonymous answers for research purpose (a specific approval was asked in the introduction text of the survey). The approach adopted in this survey was to distribute an anonymous survey, a clear statement of consent was filled by the patients that responded to the questionnaire. Therefore, the institutional review board (IRB) was not requested.

The survey consisted of 51 questions subdivided into six domains: *Socio-demographic data, Domain 1. Impact of BS on the caregiver*, *Domain 2. Quality of life of the caregiver*, *Domain 3. Role of the caregiver and Individuality*, *Domain 4. Sexuality*, *Domain 5. BS therapy*, *Domain 6. Education and awareness on BS*. The questionnaire included single choice questions and nineteen of these questions were designed using the rating scales and the Likert scale to capture the respondents’ point of view (eg. frequency: never, rarely, sometimes, often, always, etc.). Two non-mandatory open boxes where available in *Domain 1. Impact of BS on the caregiver* and on *Domain 3. Role of the caregiver and Individuality* in order to enable respondents to provide additional perspectives, since the co-design panel considered these two domains particularly important also to capture additional input from the respondents. Moreover, a specific question regarding the self-perception of being a caregiver was included; the question was represented by the following quote “*Caregiver is usually considered a person who gives care to people who need help taking care of themselves. According to this sentence, do you perceive yourself as a caregiver?*”; the respondents had the possibility to answer yes or not. After a careful discussion, the panel and the authors agreed to use, in the survey and in the presentation of the results, the word “patient”, instead of “care recipient” or “care partner” in order to ensure the understanding of the different roles and to adapt to the terminology currently in use in the BS community.

The survey was then launched nationally in Italy (and in Italian language) by means of different dissemination channels, both with the support of the patients’ association SIMBA OdV on its website, on the Facebook pages and groups of the associations, and on personal Social Media profiles of experts involved in BS (Twitter, Facebook, Instagram, etc.). The introduction of the survey clearly explained to the respondents that their answers would have been collected in an anonymous way and they also had to give their consent to the analysis of the anonymous data. The respondents answered to the questions and the survey was accessible online for three months, from the 5^th^ July 2019 to the 5^th^ October 2019.

A descriptive analysis of the information collected was performed, data are reported using number of subjects and percentage for categorical variables, mean and standard deviation for continuous variables.

## Results

### Socio-demographic profile

Ninety-four respondents answered to the survey; 55% were females and the age distribution was reported as following: 18–20 years 1%, 21–30 years 7%, 31–40 years 23%, 41–50 years 30%, 51–60 27%, 61–70 years 9%, > 70 years 3%. Most of them (62%) were partners of the BS patient, while 29% were parents, 4% son/daughter, 3% family members and 2% friends. Eighty-one percent of caregivers was married or cohabitant, 10% divorced or separated, while 9% was single; considering their education level, 73% of respondents declared to have earned at least a high school diploma, of whom academic degree or more (38%).

Regarding the profile of the patients, the mean age of the BS patients to whom caregivers were related was 49 ± 8 years; moreover, their disease duration was < 5 years in 45% of cases, between 5 and 15 years in 24%, while 31% of patients had the disease from > 15 years.

The vast majority of respondents (82%) identified themselves as caregivers, according to the definition provided in the survey.

### Domain 1. Impact of BS on the caregiver

A high percentage (65%) of the respondents have reported that they organise their life according to the needs of the patient, moreover the majority of respondents (65%) declared that BS does not affect their relationship with the patient. The answers related to the domain 2 are reported in Table 1.

**Table 1:** Domain 1 “*Impact of BS on the caregiver*”

Questions	Answers *n (%)*
*Has the illness of your family member or partner an influence on your life?*	1—My life has not been influenced at all: 7 (*7%*)
	2: 19 (*20%*)
	3: 37 (*39%*)
	4: 21 (*22%*)
	5—My life has been completely influenced: 11 (*12%*)
*Do you organise your life according to the needs of your family member or partner?*	Yes: 61 (65%)
	No: 33 (*35%)*
*Do you feel stressed of having to take care of you family member or partner?*	1—I am not at all stressed by having to take care of my family member: 31 (*33%*)
	2: 26 (*28%*)
	3: 25 (*27%*)
	4: 6 (*6%*)
	5—I am incredibly stressful by having to take care of my family member: 6 (*6%*)
*Do you think that the illness of your family member or partner affects your relationship with your friends/family/partner?*	Yes: 21 *(22%)*
	No: 61 (*65%*)
	No opinion: 12 (*13%*)
*Does the health status of your family member or partner affect your mood?*	Never: 2 (*2%*)
	Rarely: 22 (*23%*)
	Sometimes: 29 (*31%*)
	Often: 36 (*38%*)
	Always: 5 (*5%*)
*How do you evaluate your mood?*	It tends to be stable: 31 (*33%*)
	It tends to be unstable: 12 (*13%*)
	I feel depressed: 2 (*2%*)
	It tends to be anxious: 25 (*27*%)
	It tends to be calm: 11 (*12*%)
	Alterations of anxiety moments of depression: 12 (*13*%)
*How long do you work in providing care to your family member or partner (e.g. help with everyday activities or treatment management)?*	Less than 1 h per day: 58 (*62%)*
	1–2 h a day: 15 (*15*%)
	Between 2 and 4 h per day: 9 (*10*%)
	More than 4 h per day: 12 (*13*%)
*Do you feel guilty because you would like to do more for your family member or partner?*	Never: 12 (*13*%)
	Rarely: 14 (*15*%)
	Sometimes: 24 (*26*%)
	Often: 32 (*34*%)
	Always: 11 (*12*%)
*Do you have to take care of your home and family when your family member or partner does not feel okay?*	Never: 3 (*3*%)
	Rarely: 16 (*17*%)
	Sometimes: 25 (*27*%)
	Often: 23 (*24*%)
	Always: 27 (*29*%)

### Domain 2. Quality of life of the caregiver

Participants replied that they have not lost control of their life (73%) and that their attention is not completely focused on the illness (65%). The answers related to the domain 3 are reported in Table 2.

**Table 2:** Domain 2 “*Quality of life of the caregiver*”

Questions	Answers *n (%)*
*How do you generally assess your quality of life?*	1 – Better option: 9 (*10%*)
	2: 33 (35*%*)
	3: 39 (42*%*)
	4: 9 (10*%*)
	5: Worst option 3 (3*%*)
*Has your life changed since your family member or partner is ill?*	1 – My life has changed very little: 12 (1*3%*)
	2: 19 (*20%*)
	3: 27 (*29%*)
	4: 21 (22*%*)
	5 – My life has changed a lot:15 (1*6%*)
*Do you think that you have lost control of your life since your family member or partner was ill?*	Yes: 25 (27%)
	No: 69 (73%)
*Do you think your attention is completely focused on the illness of your family member or partner?*	Yes: 33 (35%)
	No: 61 (65%)
*Has your mood changed since your family member or partner was diagnosed with BS?*	1 – My mood changed very little: 24 (25*%*)
	2: 22 (*23%*)
	3: 32 (34*%*)
	4: 14 (15*%*)
	5: My mood has changed a lot 3 (3*%*)
*Has your relationship with your family member or partner changed due to the illness?*	1 – The relationship with my family member has not changed at all: 32 (*34%*)
	2: 18 (19*%*)
	3: 26 (*28%*)
	4: 13 (14*%*)
	5: The relationship with my family member has changed a lot 5 (5*%*)
*Are you afraid of your future or of the future of your family member or partner?*	Never: 7 (*7*%)
	Rarely: 9 (*10*%)
	Sometimes: 27 (*29*%)
	Often: 30 (*32*%)
	Always: 21 (*22*%)

### Domain 3. Role of the caregiver and Individuality

Most respondents (79%) considered themselves as helpful for the patient and 53% of them replied that they can freely express their emotions. Further answers related to this domain are reported Table 3.

**Table 3:** Domain 3 “*Role of the caregiver and individuality*”

Questions	Answers *n (%)*
*Do you think you can help improve the symptoms of your family member or partner?*	Yes: 47 (*50*%)
	No: 47 (*50*%)
*Would you like to play a more active role in the treatment decisions of your family member or partner?*	Yes: 38 (*40*%)
	No: 56 (*60*%)
*Do you think you are helpful for your family member or partner?*	Yes: 74 (*79*%)
	No: 19 (*20*%)
	No: opinion 1 (*1%)*
*Are you able to find moments of leisure and fun for you?*	Never: 4 (*4*%)
	Rarely: 28 (*30*%)
	Sometimes: 33 (*35*%)
	Often: 19 (20)
	Always: 10 (*11*%)
*Do you perceive that your family member or partner is asking for more help than needed?*	Never: 36 (*38*%)
	Rarely: 28 (*30*%)
	Sometimes: 17 (*18*%)
	Often: 11 (*12*%)
	Always: 2 (*2*%)
*Do you think you have enough time for you?*	Never: 2 (*2*%)
	Rarely: 28 (*30*%)
	Sometimes: 25 (*27*%)
	Often: 21 (*22*%)
	Always: 18 (*19*%)
*Do you think that your role should be better taken into account?*	Yes: 30 (*32*%)
	No: 33 (*35*%)
	No opinion 31 (*33*%)
*Do you think you can freely express your emotions?*	Yes: 50 (*53*%)
	No: 33 (*35*%)
	No opinion: 11 (*12*%)
*Do you think you need psychological support in order to better help your family member or partner?*	Yes: 23 (*24*%)
	No: 48 (*51*%)
	No opinion: 24 (*25*%)
*Would you need psychological support for yourself?*	Yes: 29 (*31*%)
	No: 46 (*49*%)
	No opinion:19 (*20*%)
*How do you generally assess the quality of life of your family member or partner?*	1 – Worst option: 12 (*13%*)
	2: 14 (*15%*)
	3: 43 (*46%*)
	4: 24 (*25%*)
	5 – Better option: 1 (*1%*)

### Domain 4. Sexuality

**Fig. 1:**
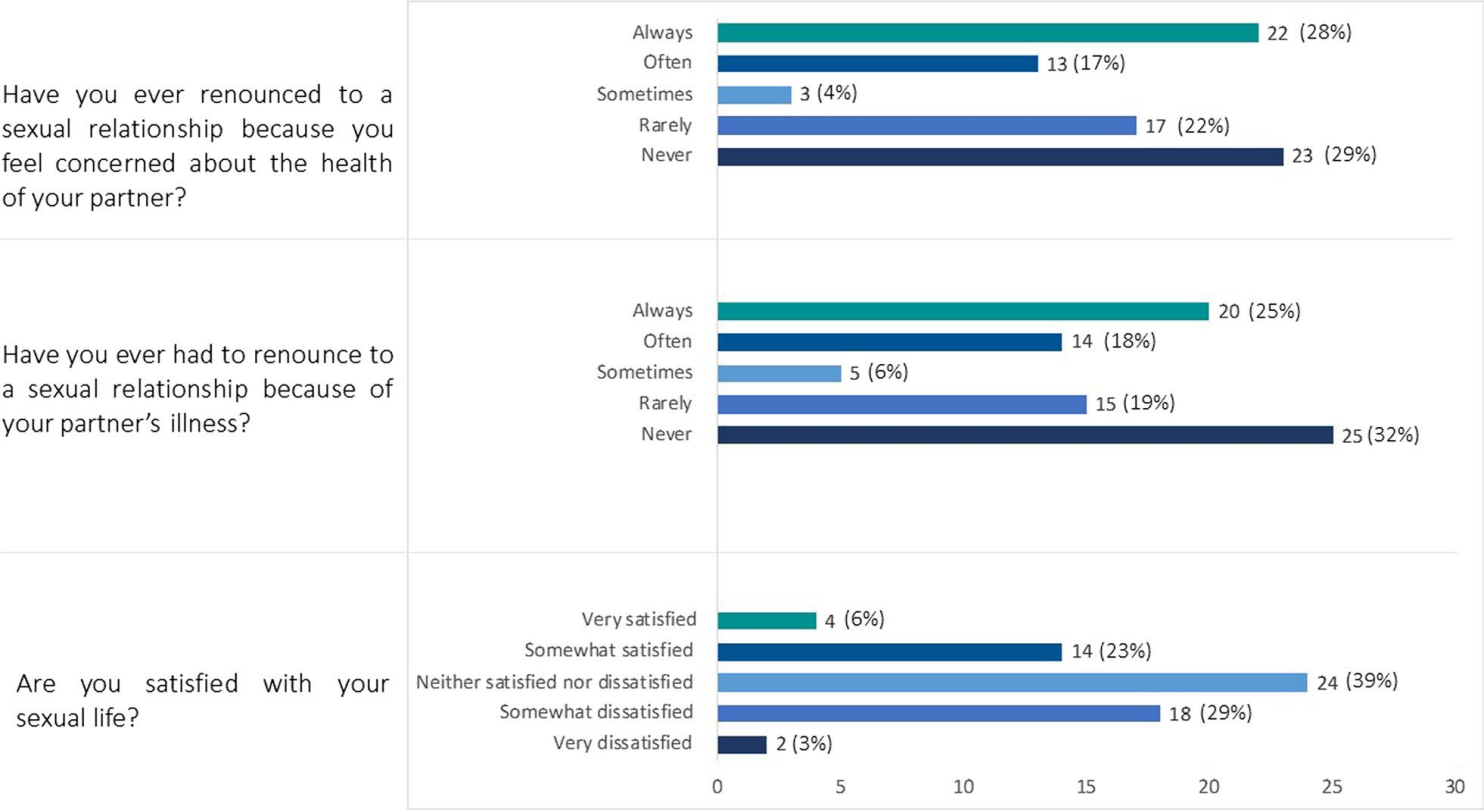
Results of the domain “Sexuality", *n (%)*

Three specific questions explored the potential impact of the disease on sexual life and the relative answers are represented in Fig. 1. Notably, 70% and 68% of the respondents reported they renounced (with a variable frequency (from always to rarely) to a sexual relationship due to concerns regarding the health of the partner or to the partner’s illness, respectively.

### Domain 5. BS therapy

**Fig. 2:**
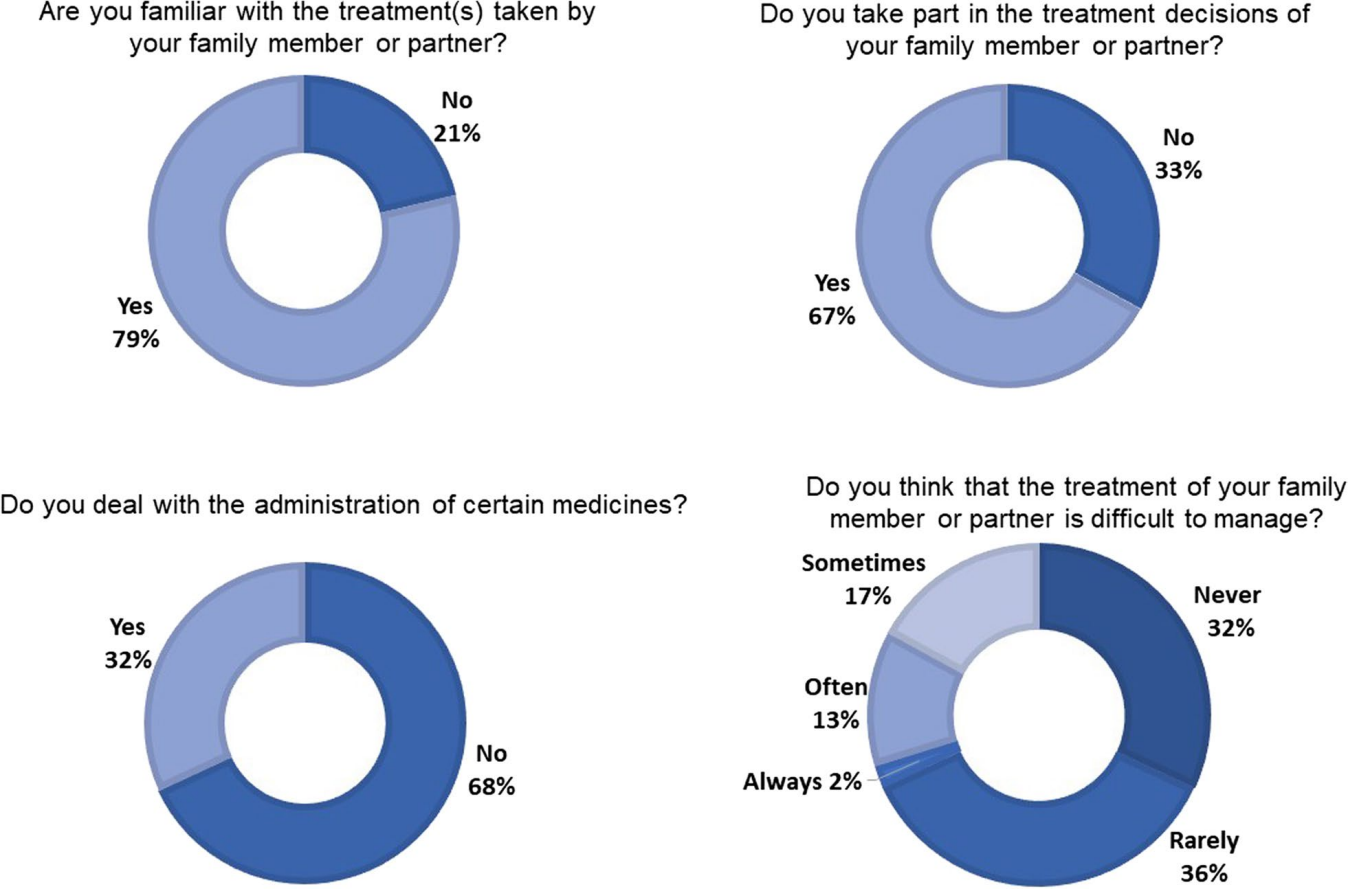
Results of the domain “BS therapy”, %

The majority (79%) of respondents indicated that they are familiar with the treatment taken by the patients and that 68% deal with the administration of some medicines. Details about questions aimed at exploring the involvement of caregiver in the therapy management and during the therapeutical decisions of the BS patient are represented in Fig. 2.

### Domain 6. Education and awareness on BS

In terms of awareness, a good percentage (64%) of respondents reported to understand the illness and, in terms of education, 68% of participants are willing to take part in training programmes dedicated to BS (see Table 4).

**Table 4:** Domain 6 “*Education and awareness on BS*”

Questions	Answers *n (%)*
*Do you think you have fully understood the illness of your family member or partner?*	Yes:60 (*64*%)
	No: 34 (*36*%)
*Do you think you are sufficiently familiar with the condition of your family member or partner?*	Yes: 52 (*55*%)
	No:21 (*22*%)
	No opinion: 22 (*23*%)
*Would you like to receive help in caring for your family member or partner?*	Yes: 28 (*30*%)
	No: 49 (*52*%)
	No opinion: 17 (*18*%)
*Would you like to take part in a training programme to learn more about the illness and treatment of your family member or partner?*	Yes: 64 (*68*%)
	No: 13 (*14*%)
	No opinion: 17 (*18*%)
*Do you know other people than your family member/friend/partner that were diagnosed with BS?*	Yes: 47 (*50*%)
	No: 47 (*50*%)
*Are you aware of the existence of a patient association for BS (SIMBA OVD)?*	Yes: 88 (*94*%)
	No: 6 (*6*%)
*Are you in contact or have you used the services of the association?*	Yes: 39 (*42*%)
	No: 55 (*58*%)
*If yes, was it useful or did you benefit from the services offered by the association?*	Yes: 71 (*75*%)
	No: 9 (*10*%)
	No opinion: 14 (*15*%)

Notably, 65% (11/17) of respondents that do not recognised themselves as caregiver are familiar with the treatment taken by his/her family member or partner in detail, and/or they deal with the administration of specific medicines.

## Discussion

The survey explored different dimensions of living with a BS patient and clearly shows how a rare disease impacts not only the patient affected by the disease, but also the life of those that are living with them and/or that contribute to their care, their informal caregivers. Informal caregivers represent important figures in the lives of people with a chronic disease, especially when the disease is rare. Their activities may range from supporting patients in scheduling appointments and helping them in the daily living matters, managing medical treatments, monitoring for signs and symptoms of disease activity or for side effects of medications and last but not least to provide emotional support [22, 23]. At some points of her/his life, someone becomes an informal caregiver; in some cases, this occurs gradually, while in others it happens really suddenly. However, specific instructions are not always in place for informal caregivers to do this ‘job’ to the best they can and above all, not always caregivers are aware of what they will be called to do to provide support to their family member or partner [24, 25]. Moreover, all the challenges are magnified when the caregivers have to deal with rare diseases, such as BS.

One of the most interesting and innovative aspects of the study was represented by the co-design process of the survey, that itself raised different central topics that the caregivers considered crucial to be explored in the questionnaire. The discussion of the questions among the members of the panel has also highly contributed to provide the caregivers with an occasion to discuss among their peers the challenges lived as partner or family member of a patient with BS. This discussion has in fact highlighted how the caregivers perceive a disease that does not have a specific phenotype and cannot always be visible or easily identifiable as BS. To this end, and from the BS patients’ perspective it is very difficult to explain not only the disease per se, but also to ensure that caregivers are able to perceive the active disease, since many symptoms are not always detectable or perceivable to them. For instance, a very common case is related to the presence of fatigue or headache that limit the patient but that cannot be visible and can therefore only be reported by the patient. This typical example of what is lived by BS patients can actually have repercussions on the caregiver, that might be willing to firstly understand the symptom(s) and then support the patient. The results of our survey suggest that the majority of our respondents think that they know the disease, but at the same a wider majority expressed the willingness of participating to educational programmes aimed knowing more about the disease and the treatments. This could be related to the fact that often caregivers are provided with some degree of knowledge and awareness on the disease, while a wider number of information, not only on the disease but also on how to support the patient – and him/herself in living with the disease should be provided to the BS caregiver. The scarcity of these empowering processes can partially explain the challenges lived by both BS patients and caregivers, in particular the need to organise their life according to the needs of the patient, how the disease affect caregivers’ mood, caregivers’ control of their own life, etc., and it can demonstrate the burning need of addressing these topics within the BS community.

The co-design process used to develop the survey has enabled the members of the panel to express the main challenges they live as BS caregivers and therefore it has ensured that the main aspects considered relevant for BS caregivers were addressed in the questions of the survey. The main dimensions emerged from their feedbacks were then translated into the domains identified in the questionnaire and into each specific question of the survey. This can already represent an important result in highlighting the need of caregivers in being considered part of the BS community and to be taken into account in the care process. The first relevant topic that was raised in the co-design panel was related to the awareness and the self-identification in the common definition of caregiver. Differently from other disease areas [26], the majority of respondents to the survey recognized themselves as BS caregivers, while less than twenty per cent did not. These results can be explained on one side by the level of awareness of the caregiver, by the actual perception of their role in the life of the BS patient and on the other side, whether these answers can be more related to the fact that some caregivers might not have fully accepted the diagnosis of the patient they live with. In fact, assessing the correlations among the questions related to the definition of caregivers and the answers on the domain of the role of the caregiver, a significant proportion of the respondents that did not identify themselves in the definition of caregiver actually replied to other questions of the domain revealing that they effectively do care for the BS patient and that can be therefore considered caregivers.

One of the most relevant topics explored in this survey is the role of the caregiver in the care and management of the BS patients. Being a chronic and relapsing disease, BS can cause debilitating and even disabling symptoms not only while the disease is active but also due to the possible impact of the long-term damages caused by the disease. The role of caregivers is for these reasons particularly important in providing support to the patient in managing everyday life activities, but also in managing their own care [12]. As reported in the open boxes of the questionnaire, the impact of BS on the life of the caregiver seems to be highly related to the need to organise his/her life according to the needs of the patient and seems to have also repercussions on the general mood of the caregiver, that often feels that he/she would like to do more and be more helpful for his/her patient. In our survey, caregivers usually spend less than one hour per day in taking care of the patient, implying that every single day they are contributing to the management of the patient, which could already be considered a burden. Caregivers can also play a crucial role in the treatment decision-making process and in our survey, in fact, the majority of respondents has indicated that they know the treatment plan of the patient in detail and has expressed their willingness in being more actively involved in the treatment decisions of the patient. The participation of the caregiver in such a crucial and central element of care might in fact contribute to increasing the adherence to the treatments, in decreasing the burden on the patients, and also in empowering caregivers in the process [27].

Besides, caregivers can provide a crucial added value also in the process related to the improvement of awareness, acceptance of the diseases, and in the daily life of the patient, providing the much needed emotional support, especially in rare and relapsing diseases such as BS. To address these important aspects, it is crucial that BS caregivers, and not only patients, are also aware of the different aspects of the disease and empowered in taking better care of themselves and of the patient they live with. On the other hand, the process of empowerment should always take into consideration the willingness and the actual availability of BS caregivers in fulfilling the role that they are expected to perform. It has to be acknowledged that BS caregivers are often expected to know the disease, the symptoms, and the treatments of BS; meeting the expectations of BS patients can already represent a burden for BS caregivers. Some might in fact not feel completely up to the expected tasks or might simply be also not in full health and have limitations in providing care and support. The contributions collected in the open boxes of the questionnaire have brought to attention and confirmed their feeling of concern towards their future ability in providing an appropriate support/care to their patient. Caregivers support might, in fact, go beyond the strict performance of tasks and might also be related to the wide definition of the role that caregivers are expected to play and these aspects should always be taken into consideration, both by the healthcare providers in providing care to the BS patients and also by policy-makers, in order to reduce the possible burden and plan possible alternatives for those patients who cannot always rely on a dedicated caregiver.

Another important aspect explored in the survey is the sexual dimension of the BS caregiver. Some caregivers that replied to our questionnaire have renounced to have sexual relationships with the partner and most of them reported that the cause of the renounce was related to the concern on the wellbeing of their partner. This new information provides interesting results on the cause of the renounce, which in BS might be generally considered to be more related with the presence of oral and/or genital ulcers (or other symptoms), while it was reported to be related to the concern of the caregiver on the general health of the patient, often mentioned as perceiving the partner as a “*fragile crystal*”.

The study was performed at national level in Italy, and it represented a first and highly needed assessment of the impact of BS on caregivers. At the same time, it included some limitations, such as the usage of a non-validated questionnaire, that was intensively discussed among the panel members. The co-design of a brand-new disease-specific survey was considered of primary importance by the panel, that expressed the need of the BS community of collecting specific information on BS caregiver, rather than general information; however, a new study is already ongoing to assess more deeply the burden of BS caregivers, adopting both validated and non-validated questionnaires. Another limitation was related to the selection of the respondents, who, due to the dissemination channels used for the survey, might be not representative of the wider BS caregivers’ community. For this reason, further studies are needed and already planned to develop disease-specific measures to explore more deeply the burden lived by caregivers as well as the possible correlations of the disease activity/severity of BS patients with the impact on the life of the caregiver. Other crucial dimensions to be further explored in future studies include the economic dimension to assess the financial burden of BS families and correlate the perspectives of BS caregivers to their socio-economic profile (level of education, working conditions, etc.) as well as to the health status of both the caregiver and the BS patient [28, 29]. These studies could highly contribute to provide a clear picture of the burden of BS caregivers and allow the identification of possible interventions and policies aimed at reducing the caregiver burden and improve the quality of life of both the caregivers and the patients living with BS.

In summary, our findings emphasize that the role of BS caregivers needs to be more acknowledged and highlighted and that there is a burning need to ensure involvement of BS caregivers, besides BS patients, in empowering processes, such as providing education and training on BS and on how to properly support themselves and the patients they live with, contributing to identifying and alleviating the BS caregiver burden.

## Conclusions

The role and importance of the caregiver is being relatively more considered in healthcare and social settings. However, within rare diseases, the role of adult caregivers is not yet fully explored, nor usually included in the assessment of the overall disease burden. The results of the co-designed survey have contributed to provide new information on BS caregivers and on their important role, as well as to identify areas of potential new initiatives for BS caregivers (and therefore patients) aimed at improving knowledge and empowering them in reducing the burden of the disease in their life and family.

## Data Availability

The data collected have been reported in the results. For further information, please contact author for specific data requests.

## References

[1] Hatemi G, Seyahi E, Fresko I, Talarico R, Hamuryudan V (2019). One year in review 2019: Behçet's syndrome. Clin Exp Rheumatol..

[2] Hatemi G, Seyahi E, Fresko I, Talarico R, Hamuryudan V (2020). One year in review 2020: Behçet's syndrome. Clin Exp Rheumatol..

[3] Talarico R, Cantarini L, d'Ascanio A, Figus M, Favati B, Baldini C, Tani C, Neri R, Bombardieri S, Mosca M (2016). Development of de novo major involvement during follow-up in Behçet's syndrome. Clin Rheumatol.

[4] Yazici Y (2020). Management of Behçet syndrome. Curr Opin Rheumatol.

[5] Hatemi G, Christensen R, Bang D, Bodaghi B, Celik AF, Fortune F, Gaudric J, Gul A, Kötter I, Leccese P, Mahr A, Moots R, Ozguler Y, Richter J, Saadoun D, Salvarani C, Scuderi F, Sfikakis PP, Siva A, Stanford M, Tugal-Tutkun I, West R, Yurdakul S, Olivieri I, Yazici H (2018). 2018 update of the EULAR recommendations for the management of Behçet's syndrome. Ann Rheum Dis.

[6] Senusi AA, Ola D, Mather J, Mather J, Fortune F (2017). Behçet's syndrome and health-related quality of life: influence of symptoms, lifestyle and employment status. Clin Exp Rheumatol..

[7] Fabiani C, Vitale A, Orlando I, Sota J, Capozzoli M, Franceschini R, Galeazzi M, Tosi GM, Frediani B, Cantarini L (2017). Quality of life impairment in Behçet's disease and relationship with disease activity: a prospective study. Intern Emerg Med.

[8] Talarico R, Palagini L, Elefante E, Ferro F, Tani C, Gemignani A, Bombardieri S, Mosca M (2018). Behçet's syndrome and psychiatric involvement: is it a primary or secondary feature of the disease?. Clin Exp Rheumatol..

[9] Talarico R, Palagini L, d'Ascanio A, Elefante E, Ferrari C, Stagnaro C, Tani C, Gemignani A, Mauri M, Bombardieri S, Mosca M (2015). Epidemiology and management of neuropsychiatric disorders in Behçet's syndrome. CNS Drugs.

[10] Talarico R, Elefante E, Parma A, Taponeco F, Simoncini T, Mosca M (2020). Sexual dysfunction in Behçet's syndrome. Rheumatol Int.

[11] Ozguler Y, Merkel PA, Gurcan M, Bocage C, Eriksen W, Kutlubay Z, Hatemi G, Cronholm PF; OMERACT Behçet's Syndrome Working Group. Patients' experiences with Behçet's syndrome: structured interviews among patients with different types of organ involvement. Clin Exp Rheumatol. 2019; 121(6):28–34 PMC9885438 31025933

[12] Brouwer WB, van Exel NJ, van de Berg B, Dinant HJ, Koopmanschap MA, van den Bos GA (2004). Burden of caregiving: evidence of objective burden, subjective burden, and quality of life impacts on informal caregivers of patients with rheumatoid arthritis. Arthritis Rheum.

[13] Torres-Made MD, Peláez-Ballestas I, García-Rodríguez F, Villarreal-Treviño AV, Fortuna-Reyna BJ, delaO-Cavazos ME, Rubio-Pérez NE. Development and validation of the CAREGIVERS questionnaire: multi-assessing the impact of juvenile idiopathic arthritis on caregivers. Pediatr Rheumatol Online J. 2020;18(1):3. doi: 10.1186/s12969-020-0400-z. 10.1186/s12969-020-0400-z PMC6961380 31937332

[14] Uzuner S, Durcan G, Sahin S, Bahali K, Barut K, Kilicoglu AG, Adrovic A, Bilgic A, Kasapcopur O. Caregiver burden and related factors in caregivers of patients with childhood-onset systemic lupus erythematosus. Clin Rheumatol. 2021 Aug 3. doi: 10.1007/s10067-021-05867-5. Epub ahead of print. PMID: 34341849. 10.1007/s10067-021-05867-5 34341849

[15] Cañedo-Ayala M, Rice DB, Levis B, Carrier ME, Cumin J, Malcarne VL, Hagedoorn M, Thombs BD; Scleroderma Caregiver Advisory Committee. Factors associated with symptoms of depression among informal caregivers of people with systemic sclerosis: a cross-sectional study. Disabil Rehabil. 2020 Feb;42(3):394–399. doi: 10.1080/09638288.2018.1500647. Epub 2018 Aug 19. PMID: 30122129. 10.1080/09638288.2018.1500647 30122129

[16] Ru J, Ma J, Niu H, Chen Y, Li L, Liu Y, Li X, Lian F, Wang X (2019). Burden and depression in caregivers of patients with rheumatoid arthritis in China. Int J Rheum Dis.

[17] Shenoi S, Horneff G, Cidon M, Ramanan AV, Kimura Y, Quartier P, Foeldvari I, Zeft A, Lomax KG, Gregson J, Abma T, Campbell-Hill S, Weiss J, Patel D, Marinsek N, Wulffraat N. The burden of systemic juvenile idiopathic arthritis for patients and caregivers: an international survey and retrospective chart review. Clin Exp Rheumatol. 2018 Sep-Oct;36(5):920–928. Epub 2018 Mar 21. PMID: 29600940. 29600940

[18] Al Sawah S, Daly RP, Foster SA, Naegeli AN, Benjamin K, Doll H, Bond G, Moshkovich O, Alarcón GS (2017). The caregiver burden in lupus: findings from UNVEIL, a national online lupus survey in the United States. Lupus.

[19] Coskun Benlidayi I, Gokcen N, Sariyildiz A, Sarpel T (2019). They have got the blues: patient's mood- and disease activity-related psychological burden of rheumatoid arthritis on caregivers. Int J Psychiatry Clin Pract.

[20] https://www.caregiver.org/resource/definitions-0/. Last access: August 2021.

[21] https://ec.europa.eu/eusurvey/home/welcome.

[22] Alfaro N, Lázaro P, Gabriele G, Garcia-Vicuña R, Jover JÁ, Sevilla J. Perceptions, attitudes and experiences of family caregivers of patients with musculoskeletal diseases: a qualitative approach. Reumatol Clin. 2013 Nov-Dec;9(6):334–9. English, Spanish. doi: 10.1016/j.reuma.2013.04.014. Epub 2013 Jul 17. PMID: 23871505. 10.1016/j.reuma.2013.04.014 23871505

[23] Rita Schriemer M, Spierings J, De Vries-Bouwstra JK, de Pundert LAJ, van den Ende CH, Vonk MC (2019). Living with systemic sclerosis: exploring its impact on caregivers. Disabil Rehabil.

[24] Stoop DF, de Vries-Bouwstra JK, Vliet TP, Vlieland (2019). Quality of life and strain among caregivers of patients with systemic sclerosis. Disabil Rehabil.

[25] Mooney J, Graham K, Watts RA. Impact of caring for someone with a rare rheumatic condition, views from patients and informal carers-the need for cat-like vigilance. Rheumatol Adv Pract. 2019 Feb 1;3(1):rkz003. doi: 10.1093/rap/rkz003. PMID: 31431991; PMCID: PMC6649 10.1093/rap/rkz003 PMC6649977 31431991

[26] Stites SD, Largent EA, Johnson R, Harkins K, Karlawish J (2021). Effects of self-identification as a caregiver on expectations of public stigma of Alzheimer's disease. J Alzheimers Dis Rep.

[27] Kelly A, Tymms K, de Wit M, Bartlett SJ, Cross M, Dawson T, De Vera M, Evans V, Gill M, Hassett G, Lim I, Manera K, Major G, March L, O'Neill S, Scholte-Voshaar M, Sinnathurai P, Sumpton D, Teixeira-Pinto A, Tugwell P, van den Bemt B, Tong A (2020). Patient and caregiver priorities for medication adherence in gout, osteoporosis, and rheumatoid arthritis: nominal group technique. Arthritis Care Res (Hoboken).

[28] Cannizzo S, Palla I, Pirri S, Triulzi I, Carta C, Taruscio D, Turchetti G (2020). One year in review 2020: economic and organisational aspects in rare and complex connective tissue diseases. Clin Exp Rheumatol..

[29] Cannizzo S, Lorenzoni V, Palla I, Pirri S, Trieste L, Triulzi I, Turchetti G (2018). Rare diseases under different levels of economic analysis: current activities, challenges and perspectives. RMD Open.

